# Beyond the pill: contraception and the prevention of hereditary ovarian cancer

**DOI:** 10.1186/s13053-022-00227-z

**Published:** 2022-06-06

**Authors:** Yue Yin Xia, Joanne Kotsopoulos

**Affiliations:** 1 Women’s College Research Institute, Women’s College Hospital, 76 Grenville Street, 6th Floor, Toronto, ON M5S 1B2 Canada; 2 Department of Pharmacology and Toxicology, University of Toronto, Toronto, ON Canada; 3 Dalla Lana School of Public Health, University of Toronto, Toronto, ON Canada

**Keywords:** *BRCA*, Ovarian cancer, Contraception, Intrauterine device, Case-control

## Abstract

*BRCA1* and *BRCA2* mutation carriers face an elevated lifetime risk of developing ovarian cancer. Oral contraceptives have been shown to significantly decrease the risk of ovarian cancer by approximately 50% in this high-risk population. Changes in contraceptive formulations and patterns of use over time have introduced lower hormonal dosages, different steroid types and non-oral routes of administration. Specifically, there has been a considerable shift in patterns of contraceptive use and the increase in the uptake of non-oral, long-acting, reversible contraception (e.g., intrauterine devices, implants, injections) has corresponded to a decline in oral contraceptive pill use. Whether or not these other methods confer a protective effect against ovarian cancer in the general population is not clear. To our knowledge, there have been no such studies conducted among *BRCA* mutation carriers. Furthermore, the impact of these changes on the risk of developing ovarian cancer is not known. In this article, we will review the existing epidemiologic evidence regarding the role of contraceptives and the risk of ovarian cancer with a focus on women with a *BRCA1* or *BRCA2* mutation. We will discuss recent findings and gaps in the knowledge while extrapolating from studies conducted among women from the noncarrier population.

## Background

The *BRCA1* and *BRCA2* tumour suppressor genes encode nuclear protein products that maintain genome integrity through various roles, including DNA repair, cell-cycle regulation and apoptosis [1, 2]. Key to their tumour suppressor activity, BRCA1 and BRCA2 are involved in homologous recombination, a high-fidelity repair pathway for DNA double-strand breaks. Although *BRCA1* and *BRCA2* are ubiquitously expressed in cells of the human body, pathogenic germline mutations in these genes confer significantly elevated risk of developing various cancers, notably, cancers of the breast and ovary [3]. The predisposition to breast and ovarian cancer specifically is not entirely understood; however, existing theories have proposed tissue-specific factors that may drive tumorigenesis at these sites including the genotoxic effects of local hormone exposure Table 1 and impaired processing of R-loops [4, 5].

**Table 1:** Summary of select studies investigating the relationship between use of a long-acting reversible contraceptive (intrauterine device, injection or implant) and ovarian cancer risk in the general population

Author, Year	Location	Type of study	Study details	Exposure	Comparison	Subgroup analysis	OR, HR, RR (95% CI)	Covariates
Tworoger, 2007 [48]	USA	Cohort	Overall (*n* = 107,900) Cases (*n* = 625) Exposed (64,847 p-years)	Any IUD	Ever use	All	1.76 (1.08–2.85)	Age, age at menarche, age at menopause, BMI, oral contraceptive use, parity, postmenopausal hormone use, tubal ligation, smoking
Wilailak, 2012 [49]	Thailand	Case-control	Cases (*n* = 330) Controls (*n* = 982)	DMPA	Ever use	All	0.61 (0.44–0.85)	Oral contraceptive use, breastfeeding, parity, family history of gynecological cancer
Urban, 2012 [50]	South Africa	Case-control	Cases (*n* = 182) Controls (*n* = 1492)	Any injectable	Ever use	All	0.69 (0.36–1.32)	Age at diagnosis, year of diagnosis, education, tobacco smoking, alcohol consumption, parity/age at first birth, number of sexual partners, urban/rural residence, province of birth
Huang, 2015 [51]	China	Cohort	Overall (*n* = 70,529) Cases (*n* = 174) Exposed (*n* = 39,054)	Any IUD	Ever use	All	0.79 (0.55–1.13)	Age, education, years of ovulation, irregular ovulatory cycles, first-degree family history of cancer, BMI, physical activity, other contraceptive methods
				Any injectable	Ever use	All	1.33 (0.58–3.04)	
Soini, 2016 [54]	Finland	Cohort	Overall (*n* = 93,843) Cases (*n* = 77)	LNG IUD	Ever use	All	0.59 (0.47–0.73)	n/a
Jareid, 2018 [53]	Norway	Cohort	Overall (*n* = 104,318) Exposed (*n* = 9146)	LNG IUD	Ever use	All	0.53 (0.32–0.88)	Age, parity, BMI, oral contraceptive use, menopausal status, maternal history of breast cancer, physical activity
Iversen, 2018 [45]	Denmark	Cohort	Overall (*n* = 104,318; 21.4 million p-years) IUD (172,265 p-years) DMPA (7321 p-years) Implant (58,371 p-years)	LNG IUD	Curent or recent exclusive use	All	0.84 (0.53–1.35)	Calendar year, parity, age, education, tubal sterilisation, hysterectomy, endometriosis, polycystic ovary syndrome, family history of breast or ovarian cancer
				DMPA	Current or recent exclusive use	All	6.56 (2.11–20.40)	
				Any implant	Current or recent use	All	0.51 (0.07–3.64)	
Wheeler, 2019 [52]	International	Meta-analysis	9 case-control studies 2 cohort studies Cases (*n* = 5133) Controls (*n* = 183,035)	Any IUD	Ever use	All	0.68 (0.62–0.75)	n/a
Balayla, 2020 [46]	International	Meta-analysis	5 case-control studies 2 cohort studies	Any IUD LNG IUD	Ever use	All	0.67 (0.60–0.74) 0.58 (0.47–0.71)	n/a
Yang, 2021 [47]	USA	Pooled analysis	NECC, NHSI, NHSII	Any IUD	Ever use	All	0.96 (0.81–1.14)	Age, body mass index, age at menarche, parity, oral contraceptive use, history of tubal ligation, family history of ovarian cancer, study center, study phase (NECC)
Phung, 2021 [44]	Australia	Pooled analysis	7 case-control studies Cases (*n* = 5064)	DMPA	Ever use	All	0.65 (0.50–0.85)	Race/ethnicity, age, education level, oral contraceptive use duration, Parity, breastfeeding

The lifetime risk of developing ovarian and/or fallopian tube cancer (referred to as ovarian cancer hereafter) has been estimated to be between 44 to 49% for women with a *BRCA1* mutation and between 17 to 21% for those with a *BRCA2* mutation [6, 7]. Given the lethality of this disease and lack of adequate screening protocols, primary prevention is strongly recommended for these high-risk women. Specifically, prophylactic bilateral salpingo-oophorectomy (i.e., oophorectomy) is advised between ages 35–40 for women with a *BRCA1* mutation and age 40–45 for women with a *BRCA2* mutation [8]. This is the gold standard for risk reduction, associated with a 80 to 96% decreased risk of cancer and a 77% reduction in all-cause mortality among women with a *BRCA* mutation [9–12].

Several hormonal and reproductive factors, including oral contraceptive use, early age at menopause, breastfeeding and parity have also been associated with a decreased risk of ovarian cancer among both women in the average-risk and high-risk population [13, 14]. Oral contraceptive use is currently the most protective, non-surgical factor among women with a *BRCA1* or *BRCA2* mutation [15–17]. Collectively, the evidence suggests that a history of oral contraceptive use reduces ovarian cancer risk by 42%, consistent with estimates observed in the general population [16]. This protective effect appears to increase with long-term use and persists after discontinuation of use [18]. In fact, the declining rates of ovarian cancer incidence and mortality observed in the United States and Europe in recent decades has been attributed to the introduction of oral contraceptives and widespread uptake since the 1960s [19–21].

Changes in contraceptive formulations and patterns of use over time have introduced lower hormonal dosages, different steroid types and non-oral routes of administration. The impact of these changes on the risk of developing ovarian cancer is not known. In this article, we will review the available observational evidence regarding the role of combined oral contraceptives as well as long-acting reversible contraceptives and the risk of ovarian cancer with a focus on women with a *BRCA* mutation. We will discuss recent findings and gaps in the knowledge while extrapolating from studies conducted among women from the noncarrier population.

### Combined oral contraceptive use and risk of ovarian cancer

Over the past five decades, the estrogen and progestin content of oral contraceptive pills has decreased rapidly in an effort to reduce the incidence of undesirable side-effects such as thrombosis and cardiovascular events without compromising contraceptive efficacy [22, 23]. Low-dose formulations are effective at suppressing ovulation [24, 25]; however, it is unclear how changes in hormonal dose and potency affect subsequent development of ovarian cancer. Available data in the general population has been mixed. An evidence report funded by the Centers for Disease Control and Prevention in conjunction with the Agency for Healthcare Research and Quality considered six case-control studies examining the effect of estrogen and progestin formulation on ovarian cancer risk [26–31]. Authors concluded that there was no relationship between the estrogen or progestin dose of oral contraceptive pills and development of ovarian cancer in the general population (odds ratio [OR] = 1.25; 95% CI 0.95–1.64 for high- vs. low-dose estrogen; OR = 0.86; 95% CI 0.60–1.21 for high- vs. low-dose progestin) [15]. In contrast, more recent data among 110,929 Nurses’ Health Study II participants suggest that women who used high-dose estrogen/high-dose progestin formulations had a non-significant increased risk of ovarian cancer compared to women who used low-dose estrogen/low-dose progestin formulations (HR = 1.34; 95% CI 0.91–1.95). This finding was likely driven by the positive association with short-term use of mestranol (HR = 1.83, 95% CI 1.16–2.88) and first generation progestins (HR = 1.72, 95% CI 1.11–2.65) compared to never users [32]. Additional studies to evaluate associations between different generations of oral contraceptives and ovarian carcinogenesis in the general population are warranted.

To our knowledge, only two reports have attempted to evaluate the impact of formulation on risk in the carrier population, using year as a proxy for hormone dose. In a retrospective cohort study of 3319 *BRCA* mutation carriers and 253 cases, Antoniou et al.*,* reported a potential increase in risk with formulations initiated after 1975, when low-dose pills dominated the market, compared to those initiated before 1975, when high-dose pills dominated the market in both *BRCA1* and *BRCA2* mutation carriers combined (HR = 1.63, 95% CI 0.95–2.81) [33]. In an updated analysis of the same cohort with additional 3115 women and 199 cases, Schrijver et al.*,* reported a significantly reduced risk of ovarian cancer for formulations initiated pre-1975 (HR = 0.57, 95% CI 0.44–0.75) and a suggestively reduced risk for formulations initiated post-1975 compared to never use (HR = 0.77, 95% CI 0.54–1.09) for *BRCA1* mutation carriers [34]. For *BRCA2* mutation carriers, formulations initiated pre-1975 trended towards a protective effect (HR = 0.69, 95% CI 0.45–1.07) whereas post-1975 use conferred a significant risk reduction (HR = 0.49, 95% CI 0.25–0.97). Both studies were limited by lack of data on the specific hormonal content of contraceptives. More research is needed to clarify the impact of hormonal dose and potency of oral contraceptives on *BRCA*-associated ovarian cancer risk.

### Overview of long-acting reversible contraceptives

In addition to oral administration, various injectable, implantable and intrauterine devices have been developed over the years for effective, long-acting reversible contraception (LARC) that require no maintenance after insertion. LARC methods can be classified into those that provide systemic delivery versus local delivery of progestin. Contraceptive implants and injections provide a continued release of progestin into the surrounding tissues that is absorbed into systemic circulation. Intrauterine devices (IUDs) provide continued local administration of levonorgestrel (LNG) to the uterus to avoid systemic effects [35]. The three LARC methods are described below with an emphasis on mechanisms of contraceptive action.

Contraceptive implants consist of progestin-only subdermal rods or capsules inserted under the skin of the upper arm. They are available as LNG systems (Norplant, Jadelle) or etonogestrel systems (Implanon, Nexplanon). Whereas etonogestrel implants consistently inhibit ovulation, about one-third of all cycles are ovulatory among users of LNG implants [36]. To maintain high contraceptive efficacy, the progestin agent confers additional local effects such as thickening of the cervical mucus and endometrial atrophy to prevent implantation in the event of a fertilized egg.

The most widely distributed contraceptive injection is depot medroxyprogesterone acetate (DMPA; Depo-Provera), which delivers a 150 mg bolus of medroxyprogesterone intramuscularly. This results in serum drug concentrations sufficient for ovulation inhibition over a period of three months [36].

An IUD utilizes a polyethylene, T-shaped frame and releases either copper or a synthetic progestin into the uterine cavity for contraceptive action. The first and most widespread hormonal IUD is the 52 mg LNG-bearing IUD (LNG IUD), commercially marketed as Mirena, Liletta or Levosert [37]. The LNG IUD is also available in 19.5 mg (Kyleena) and 13.5 mg (Skyla, Jaydess) strengths. The principle contraceptive mechanism of action of the LNG IUD is inhibition of fertilization by preventing sperm-egg union. This occurs through induction of a weak foreign body reaction, suppression of endometrial growth, and increased viscosity of cervical mucus that inhibits sperm transport [37].

Various inert and metal-coated IUDs have been historically available. The most extensively distributed non-hormonal IUD is the copper-bearing IUD (ParaGard) that contains 200 mm^2^ to 380 mm^2^ of exposed copper wire to enhance spermicidal effect. Similar to the hormonal IUD, the copper IUD inhibits fertilization by impeding sperm transport and the capacity of sperm to fertilize an ovum. The inflammatory reaction of the foreign body reaction is enhanced by the continuous release of copper ions into the luminal fluids of the genital tract [38].

### Trends in long-acting, reversible contraceptive use and impact on ovarian cancer risk

Alongside changes in the formulation and potency of oral contraception, use of LARC methods is increasing worldwide. In 2019, IUDs and oral contraceptive pills were among the most popular methods of reversible contraception globally, adopted by 17 and 16% of reproductive-aged contraceptive users, respectively [39]. From 2008 to 2014, the prevalence of IUDs and implants rose at a rate of 8.3% annually in the United States, corresponding to a decline in the use of oral contraceptive pills [40, 41]. Similar trends have been reported in Europe, where use of LARC methods increased successively between 2010 to 2013, accounting for up to 20% of contraceptive use in Sweden and 18% of that in Finland [42]. In Canada, data from British Columbia indicate that rates of hormonal IUD usage experienced the greatest growth, from 1.7% in 2006 to 6.4% in 2013 [43].

Given the growing uptake of LARC methods, it is of interest to understand whether LARCs confer a similar level of ovarian cancer risk reduction. To our knowledge, there are no studies that have reported on the relationship between use of IUDs, implants or injectables and *BRCA*-associated ovarian cancer. Available studies that have evaluated the relationships between LARCs and risk of ovarian cancer in the general population have generally suggested a mitigating effect for the use of injections and potentially contraceptive implants, while the role of IUDs is mixed [44–48].

In a recent pooled analysis of seven case-control studies by the Ovarian Cancer Association Consortium, the authors concluded a reduced risk of ovarian cancer with use of depot-medroxyprogesterone acetate (DMPA) injections (OR = 0.65, 95% CI 0.50–0.85), which is in line with the majority of other retrospective data [44, 49, 50]. Cohort studies that have explored injection use and ovarian cancer risk have showed inconsistent results. In the Shanghai Women’s Health Study, injectables were not associated with risk (HR = 1.33, 95% CI 0.58–3.04), whereas the Danish Sex Register Hormone Study observed increased risk with current or recent use of DMPA (HR = 6.56, 95% CI 2.11–20.40) [45, 51]. Both studies were limited by a low prevalence of injection users and imprecise risk estimates. With respect to subdermal contraceptive implants, only one study estimated risk of ovarian cancer among implant users in the general population [45]. The authors reported a nonsignificant inverse association between current or recent implant use and ovarian cancer risk compared to never users (RR = 0.51, 95%CI 0.07–3.64); however, any protective effect may have been underestimated because history of contraceptive use prior to study entry was not collected.

A greater number of studies have explored associations between IUD use and ovarian cancer risk in the average-risk population. Wheeler et al.*,* conducted a systematic review and meta-analysis of IUD use and the development of ovarian cancer, including nine case-control studies and two cohort studies published through 2018 [52] . Authors reported an OR of 0.68 (95% CI, 0.62–0.75) for ever use versus never use of any IUD. A significant protective effect was also observed in a more recent meta-analysis by Balayla et al. [46] A total of five case-control studies and four cohort studies were analyzed to establish an ovarian cancer risk reduction of 33% with ever use of an IUD (95% CI 0.60–0.74) [46]. Population-based cohort studies have specifically linked this protective effect to use of the hormonal, levonorgestrel-bearing IUD [53, 54]. The data with respect to nonhormonal IUDs remain controversial. A pooled analysis of data from the New England Case-Control Study and Nurse’s Health Studies reported no association between IUD use and ovarian cancer risk, wherein the majority of IUD use was attributed to nonhormonal methods [47]. Conversely, findings from the Shanghai Women’s Health Study indicated that long-term IUD use was associated with reduced ovarian cancer risk in China, where the high prevalence of IUD use was dominated by stainless steel methods [51]. Interestingly, Tworoger and colleagues observed elevated ovarian cancer risk with nonhormonal IUD use in an earlier analysis of Nurse’s Health Study, which was attributed to IUD-related peritoneal inflammation [48]. Currently, no data exists examining the relationship between IUD use and ovarian cancer risk in *BRCA* mutation carriers.

### Mechanisms of ovarian cancer risk reduction

Several underlying mechanisms may mediate the protective role of these contraceptive methods on ovarian cancer, including inhibition of ovulation and high levels of exogenous progestogens [55, 56]. Oral contraceptives, implants and injections produce sustained periods of anovulation, which is thought to eliminate the carcinogenic effects of ovulation-related inflammation and cell proliferation [57]. Oral contraceptives, implants, IUDs and injections also contain highly potent synthetic progestins that increase exogenous progestogenic exposure. Experimental data suggests that local progestogenic exposure may exert a pro-apoptotic and antiproliferative effect on transformed cells of the ovary and a necroptotic effect on abnormal cells of the tubal epithelium [58–61]. In fact, serum concentrations of DMPA are five-fold that attained by the equivalent use of oral contraceptives, which may explain the greater magnitude of risk reduction we observed with injections [44]. Progestin-mediated clearance of premalignant cells may be especially relevant for Mirena IUD users, as LNG has been measured at significant levels in the peritoneal fluid [62].

It has been hypothesized that the protective association between IUD use and ovarian cancer is related to two additional mechanisms: first, the inhibition of retrograde menstruation and second, the foreign body reaction. Hormonal IUDs are associated with decreased menstrual blood loss and amenorrhea in 20% of users [63]. This is thought to reduce retrograde flow of menstrual blood, thereby minimizing local inflammation in the peritoneal cavity and malignant transformation at the site of the ovarian surface epithelium and distal fallopian tube [64]. Finally, all IUDs elicit a foreign body reaction, a local inflammatory response characterized by the influx of immune cells to the intrauterine environment, which may allow for the elimination of occult cancer cells.

### Contraceptive use and breast cancer risk: a balancing act

The growing uptake of LARC products has raised questions about progestin exposure and the possible increased risk of breast cancer. Evidence from epidemiologic and experimental studies indicate an emerging role of progestogen signalling in breast carcinogenesis. Progesterone upregulates the nuclear factor κB (RANK)/ RANK ligand (RANKL) signalling pathway, which has been shown to drive mammary epithelial cell proliferation, mammary stem cell expansion and carcinogenesis in mice [65–67] and may be particularly relevant to breast tumor initiation in *BRCA1* mutation carriers [68–70]. In line with this understanding, we previously reported an increased risk of breast cancer with oral contraceptive use at an early age in *BRCA1* mutation carriers [71]. Specifically, women who started the pill prior to the age of 20 years had an OR of 1.45 (95% CI 1.20–1.75). This increased risk was limited to early-onset breast cancer before the age of 40 (OR = 1.40; 95% CI 1.14–1.70). For *BRCA2* mutation carriers, a nonsignificant increased risk was reported for oral contraceptive ever users based on a meta-analysis of retrospective data (OR = 1.36; 95% CI 0.89–2.10) [16]. In contrast, a recent pooled analysis of data from three large cohorts found that oral contraceptive use was not associated with breast cancer risk in the prospective analysis of *BRCA1* mutation carriers (HR = 1.08; 95% CI 0.75–1.56), although a moderately increased risk was observed in the left-truncated retrospective analysis (HR = 1.26; 95% CI 1.06–1.51) [72]. For *BRCA2* mutation carriers, oral contraceptives increased the risk of breast cancer in the prospective (HR = 1.75; 95% CI 1.03–2.97) and full-cohort retrospective models (HR = 1.52; 95% CI 1.28–1.81), but there was no association in the left-truncated retrospective analysis (HR = 1.06; 95% CI 0.85–1.33). The discrepancy between prospective and retrospective findings may indicate survival bias or a true association for younger women who were underrepresented in the prospective analysis.

Whether progestin-only contraceptives pose an increased breast cancer risk is less clearly defined. Limited data from the average-risk population is mixed regarding the association between injections and implants and the risk of breast cancer [73–75]. Although the mechanism of action of the hormonal IUD is primarily local, LNG is also released into systemic circulation with considerable interindividual variation in serum levels achieved [76, 77]. A recent meta-analysis of five case-control studies and three cohort studies concluded that the LNG IUD confers an increased breast cancer risk (OR = 1.16; 95% CI, 1.06–1.28) [78]. The role progestin-only methods on the risk of *BRCA*-associated breast cancer cannot be excluded and represents an important avenue of investigation.

Although hormonal contraception is not a replacement for preventive surgery, established guidelines conflict regarding the prescription of oral contraceptives purely for reduction in ovarian cancer risk among women with a hereditary predisposition [79–81]. The current National Comprehensive Cancer Network guidelines (2021) for the management of hereditary cancer consider contraceptive agents as a risk reduction option for high-risk women that require weighing the benefits to ovarian cancer risk alongside a potential increase in breast cancer risk [82]. Women with intact ovaries who elect for preventive bilateral mastectomy may be promising candidates for hormonal contraception with respect to cancer risk. For high-risk women who have not completed childbearing or wish to avoid surgery, contraceptive decision-making must account for her specific genetic risk, age and contraceptive needs. No studies to date have evaluated the association between LARCs and risk specifically in the carrier population and whether levels of risk reduction are equivalent to that of combined oral contraceptives warrants further investigation.

## Conclusion

*BRCA* mutation carriers face a very high lifetime risk of developing ovarian cancer, and non-invasive preventive approaches have been limited to date. The epidemiologic evidence strongly supports a significant protective effect of oral contraceptive use on the risk of developing ovarian cancer among women with a *BRCA1* or *BRCA2* mutation [13, 16, 34]. In agreement with existing recommendations, oral contraceptives should be discussed an effective contraceptive option and cancer-protective factor during the risk assessment and counselling of this high-risk population. Given the increasing popularity of injections, implants and IUDs among women of reproductive age, it is important to investigate the impact of these contemporary modes of contraception. As outlined above, studies based on women in the non-carrier population suggest that IUDs, implants and injectables similarly confer protection against disease. In the future, large observational studies with longer follow-up and detailed information on contraceptive use, including method, medication name and patterns of use, will aid to further clarify the relationship between types of contraception and the development of ovarian cancer. More experimental studies need to be conducted to delineate the mechanism of anticarcinogenic effect, particularly as it relates to IUD use.

## Data Availability

Not applicable.
